# *Heterodera glycines* utilizes promiscuous spliced leaders and demonstrates a unique preference for a species-specific spliced leader over *C. elegans* SL1

**DOI:** 10.1038/s41598-018-37857-0

**Published:** 2019-02-04

**Authors:** Stacey N. Barnes, Rick E. Masonbrink, Thomas R. Maier, Arun Seetharam, Anoop S. Sindhu, Andrew J. Severin, Thomas J. Baum

**Affiliations:** 1 Plant Pathology & Microbiology Department, Iowa State University, Ames, IA 50011 USA; 2 Office of Biotechnology, Genome Informatics Facility, Iowa State University, Ames, IA 50011 USA; 3 CHS, Inc., Grandin, ND 58038 USA

## Abstract

Spliced leader *trans*-splicing (SLTS) plays a part in the maturation of pre-mRNAs in select species across multiple phyla but is particularly prevalent in *Nematoda*. The role of spliced leaders (SL) within the cell is unclear and an accurate assessment of SL occurrence within an organism is possible only after extensive sequencing data are available, which is not currently the case for many nematode species. SL discovery is further complicated by an absence of SL sequences from high-throughput sequencing results due to incomplete sequencing of the 5’-ends of transcripts during RNA-seq library preparation, known as 5′-bias. Existing datasets and novel methodology were used to identify both conserved SLs and unique hypervariable SLs within *Heterodera glycines*, the soybean cyst nematode. In *H. glycines*, twenty-one distinct SL sequences were found on 2,532 unique *H. glycines* transcripts. The SL sequences identified on the *H. glycines* transcripts demonstrated a high level of promiscuity, meaning that some transcripts produced as many as nine different individual SL-transcript combinations. Most uniquely, transcriptome analysis revealed that *H. glycines* is the first nematode to demonstrate a higher SL *trans*-splicing rate using a species-specific SL over well-conserved *Caenorhabditis* elegans SL-like sequences.

## Introduction

Pre-mRNA splicing is a vital mechanism associated with the expression and regulation of eukaryotic genes. The most widely deployed splicing mechanism is cis-splicing, which enables the removal of intron sequences from mRNA molecules. *Trans*-splicing is less widespread and results in the fusion of RNA molecules that are transcribed from different genomic loci. The most prevalent form of *trans*-splicing involves the addition of a short, spliced leader (SL) sequence to the 5′ end of mRNA transcripts, referred to as spliced leader *trans*-splicing (SLTS). SLTS has evolved independently in a diverse set of phyla including *Nematoda, Platyhelminthes, Trypanosoma, Cnidaria, Rotifera, Chordata, Arthropoda* and *Dinoflagellata*^1–8^.

SLs originate from SL RNA genes, whose transcripts are divided into two parts by a donor splice site: a 5′ exon-like SL region and a 3′ intron-like region^9–11^. SL RNAs maintain a conserved secondary structure comprised of hairpins and a single-stranded Sm binding site (5′-purine-AU_4–6_G-purine-3), which allows the SL RNA to interact with proteins that are required for SLTS^3,12,13^.

It is evident that SLTS has a role in resolving polycistonic mRNAs in *Caenorhabditis elegans*, acting as a prerequisite for subsequent translation^14^. In *C. elegans*, approximately 70% of transcripts are *trans*-spliced to a 22nt SL: SL1 or SL2^3,15–17^. However, operon resolution is not the sole function of SLTS in *C. elegans*, as only 17% of *C. elegans* transcripts originate from operons^15,18^. It has been hypothesized that SLTS is involved in many translational regulation mechanisms, including the replacement of deleterious sequences in the 5′-untranslated region, addition of translational motifs from within the SL sequence, or by replacing a transcript’s 5′-monomethylated cap with a 5′-hypermodified cap structure^18–24^.

Sequence data indicate that all nematode species studied to date utilize SL *trans*-splicing. In all nematodes, SLs with similarity to SL1 and/or SL2 are found, with an exception of *Trichinella spiralis*, which uses non-canonical spliced leaders^25–32^. Interestingly, sequence analysis of the potato cyst nematodes *Globodera rostochiensis* and *G. pallida*, identified multiple hypervariable SL sequences in addition to SL1 and SL2^26,33^. The diversity of SL sequences found in *Globodera spp*. and the dearth of information regarding their functionality highlights a need to improve our understanding through the investigation of nematode genomic and transcriptomic data. Previous studies have identified SL1 in the soybean cyst nematode, *Heterodera glycines*, a highly damaging plant parasite closely related to *Globodera spp*.^34^. Subsequently, the SL1 sequence has been used to successfully generate *H. glycines* cDNA libraries (LIBEST_005577; unpublished McCarter, J., Clifton, S., Chiapelli, B., Pape, D., Martin, J., Wylie, T., Dante, M., Marra, M., Hillier, L., Kucaba, T. *et al*.).

In this current study, we utilize the recently assembled *H. glycines* genome^35^ and the RNA-seq reads from an early-life stages transcriptome^36^ to extensively characterize SLs and their usage in *H. glycines*. Serendipitous observation of variation in the 5′-end of two previously sequenced *H. glycines* transcripts led to the discovery of a novel SL. Through subsequent bioinformatic approaches utilizing both *H. glycines* genomic and transcriptomic data, this report shows that *H. glycines* possesses at least twenty-one SLs, found on a total of 2,532 *H. glycines* transcripts, which account for approximately one-third of *H. glycines* genes. Functional analysis of the *H. glycines* SL *trans*-spliced transcripts reveals involvement in a variety of biological processes. Interestingly, around 45% of the transcripts are promiscuously trans-spliced by SLs suggesting that there is functional redundancy amongst SL RNA molecules. Furthermore, *H. glycines* is the first nematode to show a transcriptome-wide preference for a species-specific SL sequence over the well-conserved *C. elegans* SL-like sequences.

## Results

### Discovery of a novel spliced leader in *H. glycines*

**Figure 1:**
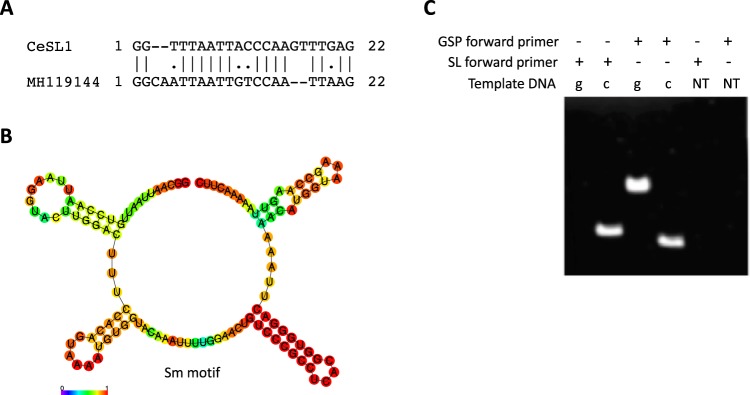
MH119144 is SL *trans*-spliced by a novel SL sequence. (**A**) Pairwise sequence alignment of *C. elegans* SL1 (CeSL1) and the first 22 nucleotides of MH119144 using EMBOSS needle with default settings. (|) = matching nucleotides, (.) = mismatched nucleotides, (−) = gap. (**B**) Secondary structural prediction of the putative MH119144 SL RNA consensus sequence using RNAfold shows a characteristic 3-hairpin SL RNA structure. (**C**) RT-PCR using *H. glycines* gDNA (g), cDNA (c) or no template DNA (NT). All lanes use a gene-specific reverse primer with either a gene-specific (GSP) forward primer or a SL forward primer.

Exploring the available *H. glycines* expressed sequence tags on NCBI revealed two transcripts coding for chorismate mutase proteins (AY160225^37^ & MH119144), which are important enzymes for parasitism in multiple plant-parasitic nematodes^37–42^. Alignment of the 5′ end of MH119144 and the SL1 primer sequence used to clone AY160225 revealed divergent 5′ ends, suggesting the presence of a novel SL sequence (Fig. 1A).

To investigate the putative MH119144 SL sequence, the entire transcript was mapped to the *H. glycines* genome with BLASTn. All but the first fifteen nucleotides of MH119144 mapped to scaffold_282 (Supplemental Fig. S1). To locate the 5′-end of MH119144 in the *H. glycines* genome, the twenty-two-nucleotide putative SL was mapped to the genome with BLASTn. The putative SL had four exact hits in the *H. glycines* genome, all of which mapped within a 2.5 Kb region on scaffold_362, confirming that MH119144 is comprised of two sequences that are located in distinctly separate regions of the genome (Supplemental Fig. S1).

In order for a SL to be functional, transcription must create a distinct non-coding hairpin SL RNA structure with a single-stranded Sm motif^3,12,13^. To identify the presence of these features, the ninety-eight nucleotides downstream of the four putative SL hits were extracted from the genome. All sequences had 99% sequence identify and displayed the typical secondary structure of functional SL RNAs (Fig. 1B).

The validity of the putative SL was tested further using RT-PCR to search for the putative SL chorismate mutase sequence in *H. glycines* gDNA and cDNA (Fig. 1C). Using the putative SL sequence as a forward primer and a gene-specific reverse primer, a visible band was produced when using a cDNA template, but not gDNA (Fig. 1C). Genic structure predictions performed on chorismate mutase indicate that the absence of a band within the gDNA reaction is not due to the primers being located on intron/exon borders. Furthermore, a control PCR amplification with cDNA and gDNA templates was performed using a gene-specific primer pair to verify the presence of the chorismate mutase gene in both DNA samples (Fig. 1C).

Collectively, three tiers of evidence support the legitimacy of this novel SL, including: mapping of the putative SL and the remainder of the transcript to separate locations within the genome, the similarity of the putative SL RNA sequence to known SL RNAs, and the absence of a SL chorismate mutase PCR product when using gDNA. This novel SL will subsequently be named *Heterodera glycines* spliced leader 3 (HgSL3) to distinguish it from *C. elegans* SL1-like and SL2-like sequences in other nematode species.

### The *H. glycines* genome contains multiple novel SL sequences

To investigate the existence of previously identified SL sequences in *H. glycines*, all known SLs from *C. elegans* and *Globodera spp*. were mapped to the *H. glycines* genome. SL1 mapped to 180 loci in the *H*. glycines genome, twenty-two sequences of which were located within close proximity to a Sm motif^13^. The only *Globodera spp*. SL variant present in the *H. glycines* genome was SL1b, which lacked a proximal Sm motif (Supplemental Table 1).

**Figure 2:**
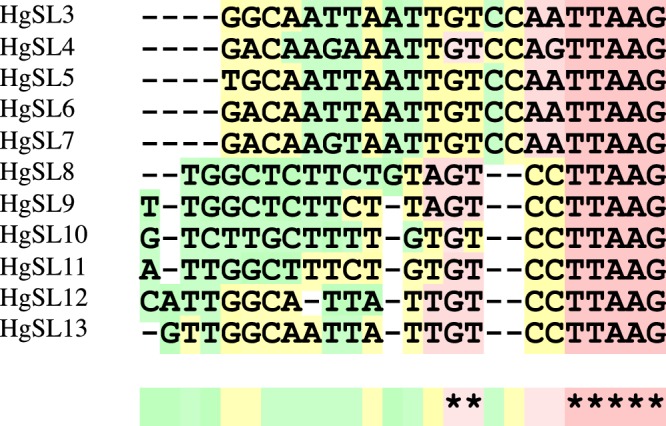
Sequence alignment of novel hypervariable *H. glycines* SLs. Multiple sequence alignment of *H. glycines* HgSL3 RNA blast results with SL RNA-like secondary structural predictions. The alignment was performed using T-coffee with default settings. (*) indicates a consensus nucleotide position and (−) denotes a sequence gap. Pink, yellow and green shading indicates good, average and bad nucleotide matches respectively.

To search for novel HgSL RNA genes, HgSL3 RNA was queried with BLAST against the *H. glycines* genome. A total of twenty sequences were identified that also contained single-stranded Sm-binding sites flanked by hairpins (Supplemental Table 1). Alignment of the first twenty-two nucleotides of the putative HgSL RNA sequences yielded ten additional unique HgSLs, numbered HgSL4–13 (Fig. 2).

### Splice leaders are promiscuously present on multiple *H. glycines* transcripts

To assess SLTS in *H. glycines*, known SLs were truncated to the 3′ most 11nt yielding a total of twenty-six unique sequences (7 from *C. elegans*, 5 from *H. glycines*, and 14 from *Globodera spp*.). The use of truncated SLs has been demonstrated to circumvent the low availability of complete 5′-ends in RNA-seq data^25,26^.

**Figure 3:**
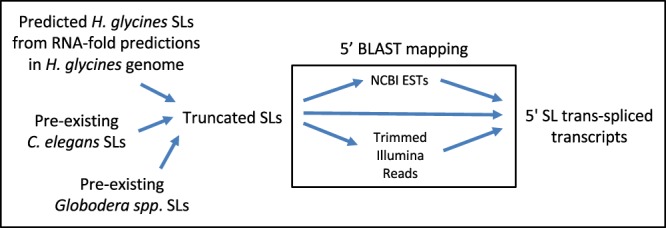
Workflow of the bioinformatic pipeline for SL trans-splicing transcript identification.

The truncated SLs were used as query sequences for three separate BLAST analyses. In the first approach, SLs were queried against the NCBI EST database, in the second approach SLs were queried against a *H. glycines* transcriptome^36^. A two-tiered third approach that involved SL queries to trimmed Illumina reads with subsequent mapping to the 5′ end of transcripts (Fig. 3). This third approach circumvents RNA-seq 5′ bias, which may result in the misassembly at the 5′ end of transcripts^43^.

**Figure 4:**
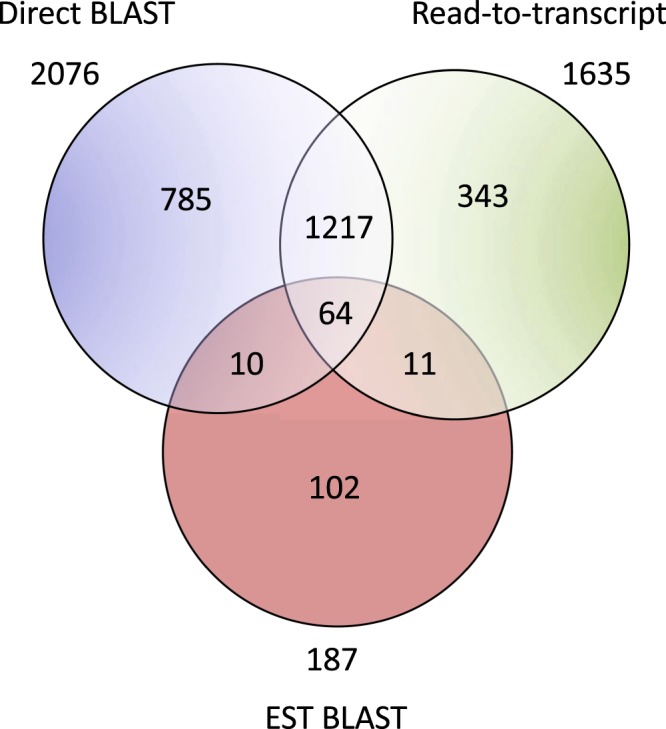
Summary of the *H. glycines* SL *trans*-spliced transcript analyses. The Venn diagram represents the overlapping and unique SL *trans*-spliced transcripts identified through the three-part approach using BLASTn against the NCBI ESTs (EST BLAST), the Trinity transcriptome (Direct BLAST) and the unassembled reads (read-to-transcript).

**Figure 5:**
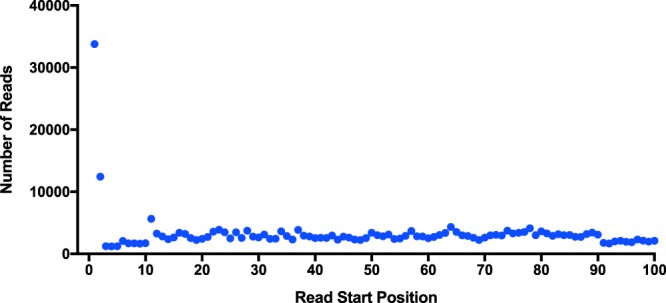
HgSLs are preferentially located at the 5′ end of *H. glycines* reads. All truncated HgSLs were queried with BLASTn against the *H. glycines* raw reads, which were also used for the Trinity assembly. The read nucleotide start positions were plotted to show the strong preference for HgSLs to be located at the 5′ end of the read.

**Figure 6:**
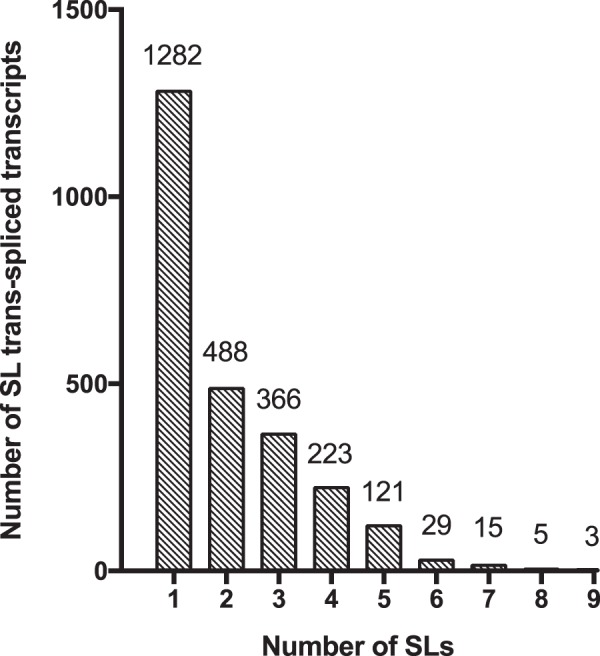
SL *trans*-spliced transcripts are promiscuously *trans*-spliced. Bars represent the number of SL-transcript combinations identified when combining the SL *trans*-spliced transcripts identified across all three BLAST analyses.

BLAST searches to ESTs and transcripts yielded 187 and 2,215 SL *trans*-spliced transcripts respectively, with a 40% (74/187) rediscovery rate of ESTs within the transcriptome (Table 1 and Fig. 4). After removing the SLs from the sequences, all ESTs were unique, while only 2076/2,215 transcripts were unique, revealing that in some cases transcripts are not uniquely spliced to one SL (Table 1 and Fig. 4). Using the read-based approach, 85,876 of ~11.4 million reads had a terminal SL, the legitimacy of which is supported by SL BLAST hits preferentially locating to the 5′-read ends (Fig. 5). Subsequent mapping of the SL-reads to the *H. glycines* transcriptome revealed a false positive rate of SL-reads at 88.4%, with 9,927/85,876 reads mapping to the 5′ end of 1,635 unique SL *trans*-spliced transcripts. Again, a portion of the transcripts appeared to be the target of more than one SL RNA molecule, resulting in 6,350 SL-transcript combinations (Table 1 and Fig. 4). Collectively, these analyses identified in 2,532 unique SL *trans*-spliced transcripts and 21 functional SLs (Table 1 and Fig. 4). Interestingly, when combining all three analyses, HgSL3 is present on 30.9% of SL *trans*-spliced transcripts making it the most abundantly used SL, a finding unique to *H. glycines*. Furthermore, 45.5% of the 2,532 SL *trans*-spliced transcripts were spliced by two or more SLs, with *trans*-splicing of five or more different SLs onto 6.8% of these transcripts (Fig. 6).

**Table 1:** Summary of genomic and transcriptomic findings for truncated *C. elegans, H. glycines* and *G. rostochiensis* SLs.

Nematode	Representative truncated SL	SL RNA in the *H.glycines* genome	Identical truncated SL members	Trinity Transcripts		
				EST BLAST	Direct BLAST	Read-to-transcript
*C. elegans*	SL1	yes	SL1b, SL1d, SL1f, SL1h	76	1267	1305
*C. elegans*	SL2.1	no	SL2.2, SL2.3, SL2.4	—	—	—
*C. elegans*	SL2.5	no	SL2.6, SL2.7, SL2.14, SL2.15, SL2.16	—	—	—
*C. elegans*	SL2.8	no		—	—	1
*C. elegans*	SL2.9	no	SL2.10, SL2.11	—	—	1
*C. elegans*	SL2.12	no	SL2.12	—	—	16
*C. elegans*	SL2.18	no		—	—	—
*H. glycines*	HgSL3	yes	HgSL5*, HgSL6*, HgSL7*,	80	899	1726
*H. glycines*	HgSL4	yes		7	6	1318
*H. glycines*	HgSL8	yes	HgSL9*	6	17	1363
*H. glycines*	HgSL10	yes	HgSL11*	—	—	—
*H. glycines*	HgSL12	yes	HgSL13*	—	—	4
*G.rostochiensis*	SL1a	no		—	—	73
*G.rostochiensis*	SL1c	no		1	1	49
*G.rostochiensis*	SL1e	no		1	—	96
*G.rostochiensis*	SL1g	no		—	1	2
*G.rostochiensis*	SL1i	no		—	—	28
*G.rostochiensis*	SL2a	no	SL2c, SL2e, SL2f, SL2g, SL2i	3	—	7
*G.rostochiensis*	SL2b	no		1	—	5
*G.rostochiensis*	SL2d	no		—	3	10
*G.rostochiensis*	SL2h	no		—	—	3
*G.rostochiensis*	SL3a	no	SL3b	—	—	2
*G.rostochiensis*	SL3c	no	SL3d, SL3e	—	—	1
*G.rostochiensis*	SL3f	no		—	—	—
*G.rostochiensis*	SL4a	no	SL4b, SL4c, SL4d, SL4e	12	21	326
*G.rostochiensis*	SL4f	no		—	—	14
				187	2215	6350

Identification of a full-length SL RNA with a proximal Sm motif within the *H. glycines* genome for each representative truncated SL is defined as yes/no in the second column. Identical truncated SL members for which a full-length sequence and proximal Sm motif were identified in the genome are denoted with an (*). The 3 methods of SL trans-spliced transcript identification and the number of SL trans-spliced transcripts identified by each method are under the collective Trinity Transcript header. EST BLAST, Direct BLAST and Read-to-transcript columns respectively indicate the number of sequences identified within the NCBI EST database, *H. glycines* transcriptome, or illumina trimmed reads that were subsequently mapped to *H. glycines* transcripts.

### Genomic features of transcripts that possess spliced leaders

To functionally characterize the genes that give rise to SL *trans-*spliced transcripts, all SL *trans*-spliced transcripts were mapped to the *H. glycines* genome using GMAP^44^. Exonic overlap between *H. glycines* genes and SL *trans*-spliced transcripts accounted for approximately one-third of the genes in the genome (9,042/29,959). It is interesting that of the 9,042 SL *trans*-spliced genes, approximately one-third (3,013) co-align with annotated repeats in the *H. glycines* genome. The ten most abundant repeats comprised 27.7% of the 3,013 *trans*-spliced genes. The most abundant functionally annotated repeat is associated with a LINE/CR1 retrotransposon (3.4%), suggesting that a significant portion of SL *trans*-spliced transcripts are associated with transposons (Table 2).

**Table 2:** Repeats associated with trans-spliced, repetitive genes.

Percent	Repeatmodeler repeat	Repeat annotation
4.82513	Motif:rnd-4_family-65	Unknown
3.68702	Motif:rnd-4_family-352	Unknown
3.36692	Motif:rnd-4_family-1273	LINE/CR1
3.09425	Motif:rnd-5_family-2862	Unknown
2.48963	Motif:rnd-3_family-228	Unknown
2.37107	Motif:rnd-4_family-1152	DNA/TcMar-Tc2
2.03912	Motif:rnd-4_family-299	Unknown
2.03912	Motif:rnd-4_family-71	DNA/Mule-MuDR
1.97985	Motif:rnd-3_family-757	DNA/MuLE-MuDR
1.82573	Motif:rnd-3_family-42	Unknown

To assess the positioning of SL *trans*-spliced genes within the *H. glycines* genome, the genome was partitioned into 50 kb bins. Analysis of the 50 kb genomic segments showed that SL *trans*-spliced genes were dispersed throughout the genome. However, clustering of SL *trans*-spliced genes was also evident, as 40 of the 2,640 50 kb bins had 14 or more consecutively arranged SL *trans*-spliced genes (Table 3).

**Table 3:** SL trans-spliced transcripts are clustered in the *H. glycines* genome.

Scaffold Number	start position	Number of consecutive SL trans-spliced genes	Scaffold Number	start position	Number of consecutive SL trans-spliced genes
6	800000	20	4	200000	15
116	100000	19	447	0	15
127	0	19	488	0	15
16	700000	19	5	250000	15
141	100000	18	9	500000	15
466	0	18	10	750000	14
5	800000	18	127	200000	14
116	150000	17	136	150000	14
68	0	17	136	50000	14
95	150000	17	154	50000	14
136	200000	16	160	0	14
192	0	16	171	0	14
2	100000	16	19	400000	14
2	150000	16	210	0	14
21	50000	16	229	0	14
10	100000	15	255	50000	14
123	50000	15	51	450000	14
127	100000	15	7	400000	14
167	50000	15	7	800000	14
345	0	15	8	900000	14

### Functional analysis of SL *trans*-spliced transcripts reveals involvement in a variety of biological processes

**Figure 7:**
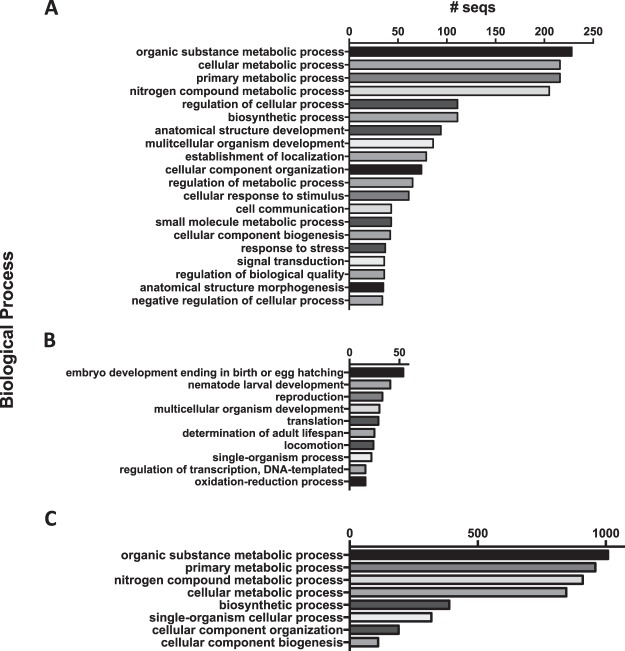
Gene Ontology (GO) biological processes for SL trans-spliced transcripts and genes. (**A**) SL trans-spliced transcripts. (**B**) Specific child terms for SL trans-spliced transcripts. (**C**) SL trans-spliced genes. Bars represent the number of proteins represented for each GO biological process that was identified using Blast2go (transcripts) or Ontologizer (genes).

In order to gain functional insight into the role of SL *trans*-splicing in *H. glycines*, the SL *trans*-spliced transcripts were annotated with Blast2go^45^ (Supplemental Table 2). Over half (52%) of the annotated transcripts were involved in metabolic and developmental processes (Fig. 7A), with the top two biological processes involved in ‘Embryo development ending or egg hatching’ and ‘Nematode larval development’ (Fig. 7B). A complementary GO enrichment analysis was performed on the corresponding genomic genes, revealing a similar profile of functions involved in metabolic processes (Fig. 7C, Supplemental Table 3).

### Effector transcripts are SL trans-spliced and display an all-or-none relationship with multi-gene copy effectors

To investigate whether spliced leaders could be involved in parasitism, we searched for exon-exon overlap between SL *trans*-spliced transcripts and effector genes in the genome. Effector genes produce proteins that are secreted by *H. glycines* during parasitism and are thought to play a major role in altering host cell structure and function. Reviewed by^46–49^. Within the *H. glycines* genome there are 80 known *bona fide* effector proteins, 28 of which originate from multiple gene copies and 51 are single gene effectors, to make a total of 121 currently confirmed effector genes. SL *trans*-spliced transcripts overlapped with the first exon of 29/121 effector genes, indicating that approximately 24% of the currently known *bona fide* effector genes are subject to SL *trans*-splicing (Table 4). Interestingly 23/28 multi-gene copy effectors display an all-or-none relationship with SL *trans*-splicing. For example, 5/5 genes corresponding to 11A06 are SL *trans*-spliced, while 0/5 genes are SL trans-spliced for 4D06 and 32E03 (Table 4, Supplemental Table 4).

**Table 4:** Effector genes giving rise to SL trans-spliced transcripts.

Effector	Total number of genes in the genome	Total number of genes that are SL trans-spliced
GLAND5	6	3
11A06	5	5
16A01	5	3
5D08	3	1
10C02	2	2
4F01	2	2
17G06	2	1
33E05	2	1
10A06	1	1
19B10	1	1
21E12	1	1
2D01	1	1
33A09	1	1
5A08	1	1
8A07	1	1
flGSB3	1	1
GLAND11	1	1
GLAND12	1	1
GLAND4	1	1

## Discussion

This study identified and functionally characterized SLs and SL *trans*-spliced transcripts of the plant-parasitic soybean cyst nematode *Heterodera glycines*. The recent availability of both the *H. glycines* genome and transcriptome has provided an opportunity to extensively characterize SL use and function in a parasitic nematode.

This study was prompted by the discovery of HgSL3 at the 5′-end of a chorismate mutase effector cDNA, leading to the identification of a unique set of hypervariable HgSLs. Novel hypervariable SLs have previously been discovered in the potato cyst nematode *G. rostochiensis* and the animal-parasitic nematode *T. spiralis*^25,26^. Interestingly, despite the high volume of SLs that have been discovered in these three species, genomic data suggest a low interspecies conservation of SLs. Given the parasitic nature of all three species, as well as the perceived link between SLs and translational regulation, it is possible that the hyper-variation of SLs is a response to parasitism of different hosts. This study investigated a possible link between SLs and known parasitic molecules, referred to as effectors, and found that 24% (29/121) of *bona fide* effector genes are subject to SL *trans*-splicing. Previous hypotheses indicate that species use SL *trans*-splicing as a form of translational control to respond to changing environments, particularly in response to nutrient availability^20^. The existence of two subsets of effector transcripts, one SL trans-spliced and one not, may provide *H. glycines* with a way to mitigate host defense responses through differentially regulating the two subsets of effectors.

To identify *H*. glycines SL *trans*-spliced transcripts, SLs were first truncated at the 5′-ends before being queried using BLAST against *H. glycines* sequences. The use of truncated SLs was previously utilized in *G pallida*^26^. Before truncating the SLs in *H. glycines*, we first verified that this approach was necessary by using the full-length SLs as query sequences against the *H. glycines* ESTs and transcriptome. Only fifteen sequences, none of which were SL1, were identified across both databases (available in GitHub). The failure to recover full-length SL1 supports the lack of 5′-ends within the *H. glycines* datasets, as SL1 is present in *H. glycines* and other related nematodes^26,27^. The read-based approach further verified the lack of complete 5′-ends within the *H. glycines* transcriptome by showing that the truncated SLs were predominantly located at the first nucleotide of raw reads that were underrepresented in mature transcripts. To further complicate transcriptome assembly in SLTS organisms, this study revealed that 45.5% of SL *trans*-spliced transcripts do not have one unique SL-transcript combination. The promiscuous nature of SLs on otherwise identical transcripts may cause high ambiguity in the assembly step, resulting in 5′ truncations or the assembly of a transcript that reflects only the most abundant SL-transcript while discarding lower expressed SL-transcripts.

Analysis of the available *H. glycines* ESTs, transcriptome and raw reads used in this study concluded that HgSL3 is the most prevalent SL in *H. glycines*, with 30.9% of the SL *trans*-spliced transcripts being *trans*-spliced by HgSL3. The predominant use of a non-SL1 sequence is unique to *H. glycines* and contrasts with findings in *C. elegans* and the animal-parasitic nematode *Ascaris suum*, as well as *G. pallida* where SL1 and SL1 variants were identified on >90% of the SL-containing *G. pallida* reads^26^.

*C. elegans* operon genes, which are resolved into monocistronic transcripts using SL *trans*-splicing, are upregulated during recovery from growth-arrested states^14,50^. Operon arrangement is believed to be advantageous in *C. elegans* during times of limited resources as there are less promoters competing for transcriptional resources^50^. In the case of *H. glycines*, SL *trans*-spliced transcripts were found to be involved in ‘Embryo development ending or egg hatching’ and ‘Nematode larval development,’ suggesting that SL *trans*-splicing may also be involved in initiating developmental changes in *H. glycines*. Operon arrangement has not yet been defined in *H. glycines*, however the clustering of SL *trans*-spliced transcripts in the genome suggests the presence of operon-like structures.

To both adapt and improve upon existing SL identification pipelines^18,51,52^, we developed a SL identification pipeline that utilizes generic RNA-seq, assembled transcripts, and ESTs, rather than requiring SL trapping prior to sequencing^53,54^. This method provides an alternative to existing pipelines by utilizing the propensity for SLs to be trans-spliced at 5′ ends and avoiding the requirement of unmapped reads having dual genomic mapping^18,51^. Additionally, using both pre-existing and predicted SL sequences that follow canonical SL RNA structures, we allow for the identification of novel SLs. A drawback of this approach may lie in the requirement of SLs to reside at 5′ ends, which is reliant on accurate adaptor trimming and prior knowledge of the anticipated SL length.

In summary, *H. glycines* possesses a unique set of hypervariable SLs, which are promiscuously *trans*-spliced to the 5′ end of >2,000 *H. glycines* transcripts, equivalent to approximately one-third of *H. glycines* genes. A robust identification of SLs was possible through novel methodology and the availability of *H. glycines* genome and transcriptome sequences. As more data becomes available for *H. glycines* and other parasitic and non-parasitic nematodes, the functional significance of SLTS may become more apparent and potentially lead to novel control measures.

## Materials and Methods

### Identification and structure prediction of putative SLRNAs

All *G. rostochiensis*^26^, *C. elegans* (PRJNA13758) SL sequences and the 22nt SL sequence from MH119144 were queried to the *H. glycines* genome with BLASTn V2.4.0 + (E-value 1.0e-3)^55^. SL hits and the adjacent 3′ 98 nucleotides were extracted using Samtools V1.4^56^. Secondary structure was predicted using RNAfold V2.1.9 with unpaired bases participating in at most one dangling end. All extracted sequences were analyzed for a downstream Sm motif (5′-purine-AU_4–6_G-purine-3′)^57^.

### DNA extraction and amplification

To confirm the functionality of putative SL on transcript MH119144 OP50 *H. glycines* was propagated on Williams 82 soybean. To isolate mixed-stage nematodes, root tissue was macerated with a blender, sieved and separated with a sucrose gradient^58^. Nematodes were ground in liquid nitrogen and total RNA was extracted using a RNeasy Mini Kit (Qiagen, Valencia, CA, USA). One μg of total RNA was treated with DNase I (Thermo Fisher Scientific, Waltham, MA, USA) and cDNA was synthesized using qScript cDNA SuperMix (Quantabio, Beverly, MA, USA). Genomic DNA was also extracted from ground nematode tissue using QIAamp DNA Mini Kit (Qiagen). RT-PCR was performed on a Bio-Rad S1000TM thermal cycler with reactions containing 1X PCR buffer, 1.5 mM MgCl2, 0.2 mM dNTP, and 1 unit of Taq DNA Polymerase (ThermoFisher Scientific). Thermocycler conditions were: 94 °C for 3 min, 35 cycles of 95 °C for 45 s, 55 °C for 30 s and 72 °C for 1 min, followed by 10 min at 72 °C.

### Identification of SL *trans*-spliced transcripts

All putative SL sequences were queried with BLASTn to the *H. glycines* NCBI EST database, referred to as EST BLAST^55,59^ and a *H. glycines* de novo Trinity transcriptome assembled from NCBI SRA accession SRP122521, referred to as Direct BLAST^36^. BLAST hits were filtered to within the first thirteen nucleotides of the transcript, and with a 10nt minimum alignment length. ESTs and trinity transcripts were mapped to the genome using GMAP 20170317, and transcript to gene relationships were identified using exon to exon overlaps with Bedtools intersect V2.26.0^44,60^. Genes were clustered by location using custom bash scripts.

### Read Analysis

In the method referred to as Read-to-transcript, SLs were truncated to the 11 3′ nucleotides and queried with BLASTn V2.4.0+ to Sickle-trimmed (default)^61^, paired-end reads (word_size 5, -dust no, -task blastn-short) used in generating a *H. glycines* trinity transcriptome^55^. The subject start position for hits was graphed using GraphPad Prism 4. BLAST output was filtered by a 10 bp minimum alignment length and hits within 12 bp of the appropriate read end. Putative SL-containing reads were queried with BLASTn V2.4.0+ to the transcriptome and filtered by 80 bp min alignment length, and within 12 bp of the 5′ transcript end^55^.

### Functional Analysis

Functional annotation was performed using Blast2go V4.1. All transcript sequences were searched against the NCBI NR database using Blastx (e-value 1.0–5. Interpro scan was performed using all default applications, and sequences were annotated with an annotation cutoff of 55 and a GO weight of 5^45,62^. GO enrichment for *trans*-spliced genes was performed using Ontologizer V2.0 with gene functions from the *H. glycines* genome^63^.

### *Trans*-splicing in Effector and Repetitive Genes

Bedtools intersect V2.26.0 and custom bash scripts were used to identify *trans*-spliced repetitive genes from a Repeatmodeler V1.0.8 tracks of the genome^60,64^. Effector genes were mapped to the genome using GMAP 20170317^44^, and were subjected to bedtools intersect V2.26.0 and custom bash scripts to identify *trans*-splicing effectors^60^.

## Supplementary information

Figure S1

Dataset 1

Dataset 2

Dataset 3

Dataset 4

## Data Availability

The *H. glycines* expressed sequence tags analyzed during the current study are available through the National Center for Biotechnology Information website. The *H. glycines* transcriptome data is from the publication 10.1038/s41598-018-20536-5. The *H. glycines* genome is publically available on the SCNbase website (https://www.scnbase.org). All scripts used for bioinformatic analysis are available at (https://github.com/ISUgenomics/Heterodera-glycines-Spliced-Leaders/blob/master/StaceyBarnesRestart.md).
