# Supplementary material for: Heterodera glycines utilizes promiscuous spliced leaders and demonstrates a unique preference for a species-specific spliced leader over C. elegans SL1
Source: Sci Rep. 2019 Feb 4;9:1356. doi: 10.1038/s41598-018-37857-0 (PMC6362198; doi:10.1038/s41598-018-37857-0)
Supplement: Supplementary file 1 — Figure S1 [file 41598_2018_37857_MOESM1_ESM.pdf]

***Heterodera glycines* utilizes promiscuous spliced leaders and demonstrates a unique preference for a species-specific spliced leader over *C. elegans* SL1**

Stacey N. Barnes[1], Rick E. Masonbrink[2], Thomas R. Maier[1], Arun Seetharam[2], Anoop S. Sindhu[3], Andrew J. Severin[2], Thomas J. Baum[1*]

[1]Plant Pathology & Microbiology Department, Iowa State University, Ames, IA 50011, USA
[2]Office of Biotechnology, Genome Informatics Facility, Iowa State University, Ames, IA 50011, USA
[3]CHS, Inc., Grandin, ND 58038, USA

Author Contact Information

Stacey N. Barnes – snbarnes@iastate.edu

Rick E. Masonbrink - remkv6@iastate.edu

Thomas R. Maier - trmaier@iastate.edu

Arun Seetharam - arnstrm@iastate.edu

Anoop S. Sindhu - anoopsindhu@gmail.com

Andrew J. Severin - severin@iastate.edu

*Thomas J. Baum – tbaum@iastate.edu

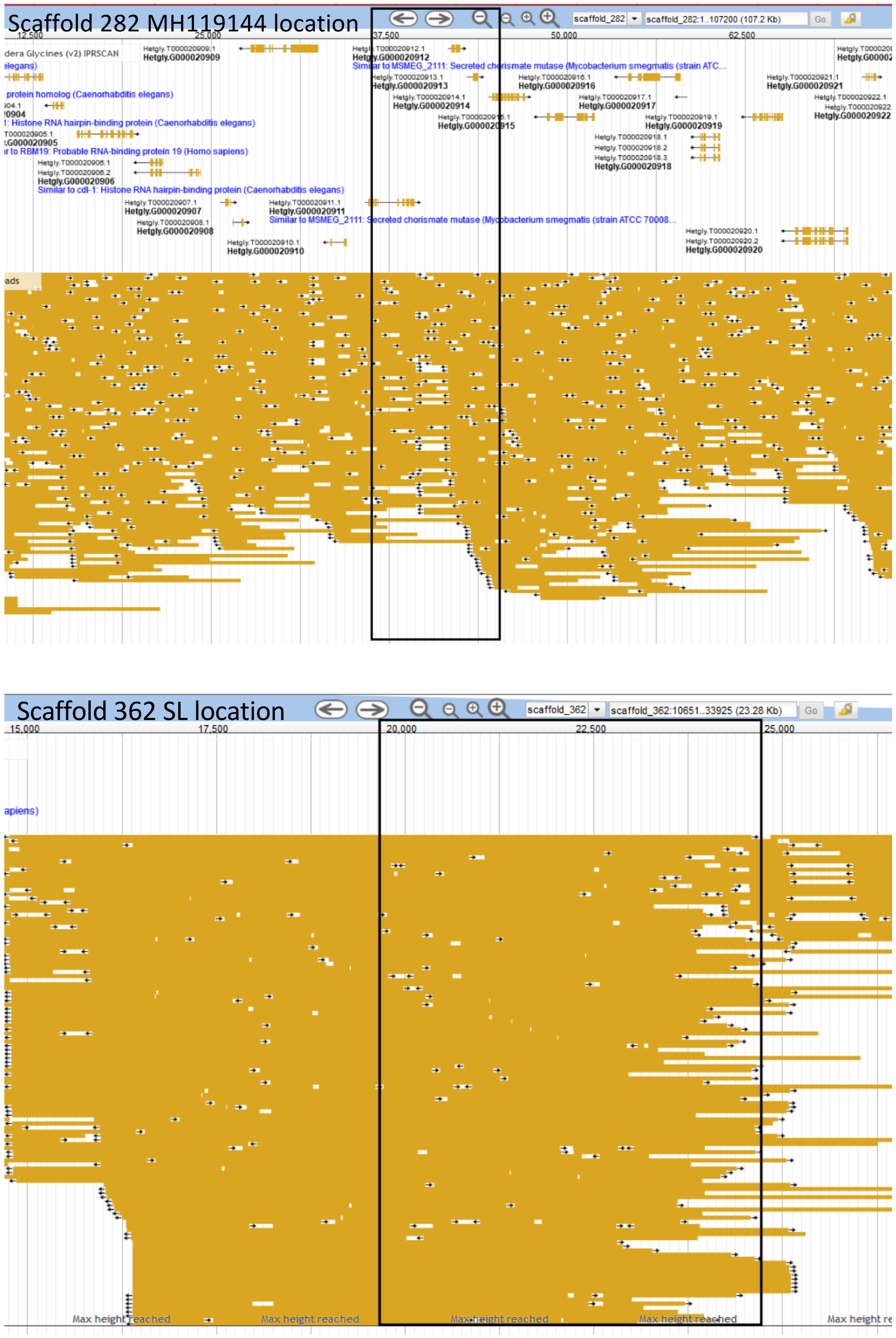

Supplementary Fig. S1. BLAST hits for MH119144 are located within well supported regions of the *H.* glycines genome. Alignment of pacbio subreads are indicated with yellow lines and the regions corresponding to BLAST hits for MH119144, excluding the first 15nt (top panel), and the putative SL sequence, nucleotides 1-15 (bottom panel) are indicated by a black box.
